# Weather-based prediction of *Plasmodium falciparum *malaria in epidemic-prone regions of Ethiopia II. Weather-based prediction systems perform comparably to early detection systems in identifying times for interventions

**DOI:** 10.1186/1475-2875-3-44

**Published:** 2004-11-19

**Authors:** Hailay D Teklehaimanot, Joel Schwartz, Awash Teklehaimanot, Marc Lipsitch

**Affiliations:** 1 Department of Epidemiology, Harvard School of Public Health, 677 Huntington Avenue, Boston MA 02115, USA; 2 Department of Environmental Health, Harvard School of Public Health, 677 Huntington Avenue, Boston MA 02115, USA; 3 Mailman School of Public Health, Columbia University, New York, NY, USA

## Abstract

**Background:**

Timely and accurate information about the onset of malaria epidemics is essential for effective control activities in epidemic-prone regions. Early warning methods that provide earlier alerts (usually by the use of weather variables) may permit control measures to interrupt transmission earlier in the epidemic, perhaps at the expense of some level of accuracy.

**Methods:**

Expected case numbers were modeled using a Poisson regression with lagged weather factors in a 4^th^-degree polynomial distributed lag model. For each week, the numbers of malaria cases were predicted using coefficients obtained using all years except that for which the prediction was being made. The effectiveness of alerts generated by the prediction system was compared against that of alerts based on observed cases. The usefulness of the prediction system was evaluated in cold and hot districts.

**Results:**

The system predicts the overall pattern of cases well, yet underestimates the height of the largest peaks. Relative to alerts triggered by observed cases, the alerts triggered by the predicted number of cases performed slightly worse, within 5% of the detection system. The prediction-based alerts were able to prevent 10–25% more cases at a given sensitivity in cold districts than in hot ones.

**Conclusions:**

The prediction of malaria cases using lagged weather performed well in identifying periods of increased malaria cases. Weather-derived predictions identified epidemics with reasonable accuracy and better timeliness than early detection systems; therefore, the prediction of malarial epidemics using weather is a plausible alternative to early detection systems.

## Background

Malaria epidemics are reported frequently and have caused high morbidity and mortality among all age groups in the African highlands [1-4]. Early detection and accurate forecasting of the time, place and intensity of these epidemics is important for emergency preparedness, planning and response [5,6]. Considerable efforts are being made to promote, develop and implement early warning systems for malaria epidemics in Africa [5,7]. Ideally, public health and vector control workers would have access to a system that alerts them when substantial numbers of excess cases are expected, and such alerts should be sensitive (so that alerts are reliably generated when excess cases are imminent), specific (so that there are few "false alarms") and timely (so that there is adequate lead time to act). Generally, each of these performance characteristics is enhanced at the expense of another. The value of interventions – such as larviciding, residual house spraying and mass drug administration – to control malaria epidemics has been documented [8]. However, due to the explosive nature of malaria epidemics, the usefulness of such interventions in epidemic settings depends on timely information about the onset of a severe epidemic.

Early detection systems, which are used to detect epidemics once they have begun, can correctly identify periods that are defined by expert observers as "epidemic," albeit with varying specificity. A number of such systems have been proposed or implemented. For example, WHO has advocated the use of alerts when weekly cases exceed the 75^th ^percentile of cases from the same week in previous years [9] and other methods, based on smoothing or parametric assumptions, have also been considered [10-12]. However, an early detection system, which generates alerts once unusually high case numbers are already observable, will be useful for targeting interventions only if it identifies epidemics at an early phase, when there is still time to take effective action [13] and if the epidemics persist (and indeed grow) over time so that action taken after the warning can still have an effect. It was previously shown, using weekly case numbers from 10 districts in the Ethiopian highlands over approximately 10 years, that simple weekly percentile cutoffs used for early detection are capable of identifying periods with unusually high malaria incidence, and that interventions that take effect within two weeks of such alerts could have a substantial impact in reducing excess cases [14].

While early detection systems appear to provide timely information about the onset of severe epidemics, they intrinsically trigger alerts only when unusual transmission is already underway. Another approach, known as "early warning," attempts to predict epidemics before unusual transmission activity begins, usually by using weather variables that predict vector abundance and efficiency, and therefore, transmission potential [6,15-18]. The advance notice provided by an early warning system could allow action to be taken earlier in the course of the epidemic, or could increase the span of time available to undertake control measures before the predicted excess cases occur. A number of authors have used weather factors to attempt to predict malaria epidemics [19-24], and Teklehaimanot et al. [25] showed that polynomial distributed lag (PDL) models incorporating lagged effects of minimum temperature, maximum temperature and rainfall could mimic seasonal patterns of malaria incidence in the same ten sites for which early detection algorithms were evaluated. Because the significant weather predictors of malaria cases are lagged by four or more weeks, such prediction systems may, in principle, provide a means of anticipating unusual malaria incidence with more lead time than early detection methods.

Here, an attempt to combine these avenues of previous work is described, using modified versions of previously described models based on weather factors to provide predictions of Plasmodium falciparum cases in these 10 districts of Ethiopia, and evaluating thresholds that trigger warnings. The hypothesis tested here is that the use of predicted cases (rather than actual cases, as in our previous work on early detection) would reduce the precision of the alert thresholds (resulting in alerts whose timing was less well matched to periods of excess cases than those generated by early detection), as the price of obtaining the alerts with greater advance notice. In fact, the early warning system based on predicted cases performed slightly worse in most cases than the early detection system, but the performance was rarely much worse and occasionally slightly better. These comparisons are described and their implications for the choice of malaria prediction/detection systems in epidemic-prone areas of Africa are discussed.

## Methods

### Study area and data

Microscopically confirmed malaria cases were collected from a health facility in each of ten districts of Ethiopia over an average of 10 years; this data set has been previously described [14]. Each of these health facilities serves people living in the surrounding localities with few exceptions coming from other places. The data were extracted (by species) from records of outpatient consultations for the years 1990 through 2000. The analysis was restricted to *P. falciparum*. The original data collected on the basis of Ethiopian weeks (where the number of days in each week varies between 5 and 9) were normalized to obtain mean daily cases for each Ethiopian week [14].

Daily meteorological data (minimum and maximum temperatures and rainfall) recorded at the local weather stations nearest to the health facility were obtained from the National Meteorological Services Agency (NMSA) for the same period. These daily data were collapsed into weekly data to correspond with the weekly malaria cases. The weekly mean for minimum and maximum temperatures and the total weekly rainfall were calculated from the daily records.

#### 1) Modeling the relationship between predictors and malaria cases

The expected case numbers for a given week were modeled using a Poisson regression with lagged weather factors, an autoregressive term, a time trend and indicator variables for week of the year. Biological considerations about the interrelationship between weather, mosquito and malaria parasite suggest that malaria cases should follow periods of increased temperature and increased rainfall, at defined intervals [26-28]. Thus, lags of 4 – 12 weeks for rainfall, and 4 – 10 weeks for minimum and maximum temperatures were considered [25]. In addition week and time trend, as well as an autoregressive term (based on a moving average of the number of cases four, five and six weeks before) were included, which is intended to improve the prediction. Because of the Poisson regression context the autoregressive term enters logarithmically. A 4^th^-degree polynomial distributed lag (PDL) model [29] was fitted to the data. This reduces the number of degrees of freedom for each weather factor from the number of lags considered and circumvents some of the difficulties associated with estimation of coefficients for multiple lags, including instability of estimates due to collinearity of the different lags of the same variable. The generalized form of the model is thus expressed as:



where *E*(*Y_st_*) denotes expected value for the daily average number of malaria cases at site *s *on week *t*; , , *R_t-i_*, and *Y_st-i _*are the weekly minimum and maximum temperatures, rainfall and autoregressive term *i *weeks previously; *t_s _*and *W_s _*designate time trend and week in a year at site *s*; *α_s _*represent the intercept, at site s.

#### 2) Epidemic Prediction Strategies

For each week at each location in the data set, the number of cases was predicted using equation (1) and data available four weeks prior to the week for which the prediction is made. Coefficients of equation (1) were obtained using all years except that for which the prediction was being made, to avoid circularity. The prediction for week t was then made using this all-but-current-year model with weather and case data for the weeks up to week t-4. The predicted number of cases is thus estimated using the following model:



where  represent the predicted cases for year j; , , , , ,  and  are parameter estimates (for intercept, minimum and maximum temperature, rainfall, time, week and autoregressive term respectively) from all years except j; , , , *t^j^*,  and  are minimum and maximum temperatures, rainfall, time, week and autoregressive term respectively from year *j*.

#### 3) Evaluation of the prediction system

##### Expected number of cases to be used as threshold levels

In early detection algorithms, actual cases in a given time period are typically compared against some threshold level of cases to determine whether excess cases have been observed. Often, the threshold level represents an upper bound on "normal" case numbers from previous years. If this threshold level is crossed (perhaps, depending on the system, for several consecutive weeks), an alert is generated [14]. Such systems for early detection have been evaluated previously [14].

In this study of the usefulness of prediction systems for generating alerts, historically based thresholds were similarly used – weekly percentile (defined as a given percentile of the case numbers obtained in the same week) or weekly mean with standard deviation (defined as the weekly mean plus a defined number of standard deviations) algorithms as threshold levels [14] – but generated alerts when predicted cases for a week exceeded the threshold. The thresholds for each year were calculated on the basis of all other years in the data set for a given health facility, excluding the year under consideration. In each case, an alert was triggered if the defined threshold was exceeded by the predicted number of cases for two consecutive weeks (this choice is intended to improve the specificity of the alert system for any given threshold). If another alert was triggered within six months, it was ignored, on the assumption that intervention following the first alert would prevent another epidemic within the next six months. Algebraic descriptions of the thresholds are given below:

1. Weekly percentile

Threshold is exceeded when , where *T_sij _*= *Q_psij_*, where *Q_psij _*represents the *p*th (*p *= 70, 75, 80, 85, 90, or 95) percentile of observations from week *i *at facility *s *in years other than *j*.

2. Weekly mean with standard deviation.

Threshold is exceeded when , where *T_sij _*= *μ_sij _*+ *βσ_Ysij_*, where *β *= 0.5, 1.0, 1.5, 2.0, 2.5 or 3.

### Measure of performance of each alert

The effectiveness of alerts generated by our four-week-ahead prediction system was compared against that of alerts based on a detection system using actual cases. Since the prediction system generates predicted numbers of cases four weeks ahead of time, this permits implementation of control measures four weeks earlier than under a detection system. On the other hand, one would expect that the accuracy of prediction might be less than that of detection. The comparisons were designed to assess this trade-off between the ability to act earlier in possible epidemics and the possible loss of accuracy.

A method previously described [14] was used to compare different alert-generating procedures on a scale that reflects their operational uses. Briefly, this method quantifies the usefulness of a particular alert-generating system set to a given sensitivity by estimating how many malaria cases might be prevented by measures taken after each alert generated by the system, with defined assumptions about the lead time from alert to the effectiveness of such measures, and about the duration of effectiveness of these measures. Potentially prevented cases (PPC) for each alert are defined as a function of the number of cases in a window following the alert. To obtain the PPC, the following three assumptions were made. (a) It was assumed that four weeks elapse from the decision to make an intervention based on an alert until the interventions take effect. (b) From that time, the window of effectiveness is assumed to last either eight or 24 weeks (to account for control measures whose effects are of different durations). (c) Since no control measure would be expected to abrogate malaria cases completely, two possibilities were considered for the number of cases in each week of the window that could be prevented: 1) cases in excess of the seasonal mean (low effectiveness) and 2) cases in excess of the seasonal mean minus one standard deviation (high effectiveness). These different assumptions allowed testing the sensitivity of the performance of the prediction and detection systems to the length of the window of effectiveness and the choice of function to define potentially prevented cases. When the observed number of cases in a week is less than the seasonal mean or the seasonal mean minus the standard deviation, PPC is set to a minimum value of zero for that week.

### Methods of comparison

For each value of each type of threshold (applied to either the predicted and observed number of cases) at each health facility, the number of PPC was transformed into a proportion (percentage), by adding the number of PPC for the alerts obtained and dividing this sum by the sum, over all weeks in the data set, of the number of potentially prevented cases. Proportion rather than actual cases were used because the numbers of malaria cases vary from district to district. To compare the performance of the predicted and observed cases on a single scale, a curve was plotted for each algorithm showing the mean percent of PPC (%PPC) over all districts versus the average number of alerts triggered per year, with each point representing a particular threshold value. Better methods of generating a warning were those that potentially prevent higher numbers of malaria cases using smaller numbers of alerts.

### Random and optimally timed alerts

The performance of the alerts provided by both the predicted and observed cases was compared with random and optimally timed alerts. PPC was estimated for alerts chosen on random weeks during the sampling period. To estimate the performance of optimally-timed alerts (which could not have been implemented but is optimal in hindsight), the optimal timing of alerts were identified by retrospectively going through data if one had perfect predictive ability; the optimal week for one alert was chosen; then by going through the remaining weeks, the optimal week for a second alert was chosen, and so on. The optimal alert would serve as an upper bound curve for the best choice of alert times, given a defined alert frequency [14].

### Cold versus hot districts

The relative importance of weather factors in determining malaria transmission significantly depends on the climate of the area. It has recently been shown that although rainfall was significantly associated in cold and hot districts, minimum temperature contributed only in the cold districts of Ethiopia [25]. Furthermore, Zhou et al. [30] showed that there was high spatial variation in the sensitivity of malaria outpatient numbers to climate fluctuations in East African highlands. To determine the effect of the differential contribution of weather factors on the accuracy of predictions, the performance of predictions in the hot and cold environments were compared. Thus, districts with similar climatic characteristics (on the basis of altitude and temperature) were grouped, in order to produce more generalizable results within similar climatic conditions. The hot districts (altitude < 1700 mm above sea level) included Diredawa, Nazareth, Wolayita and Zeway; and the cold districts included Alaba, Awasa, Bahirdar, Debrezeit, Hosana and Jimma. Mean %PPC and the average number of alerts for the cold and hot districts were obtained and the same method was used to compare the performance of the prediction system in the hot and cold districts.

## Results

**Figure 1:**
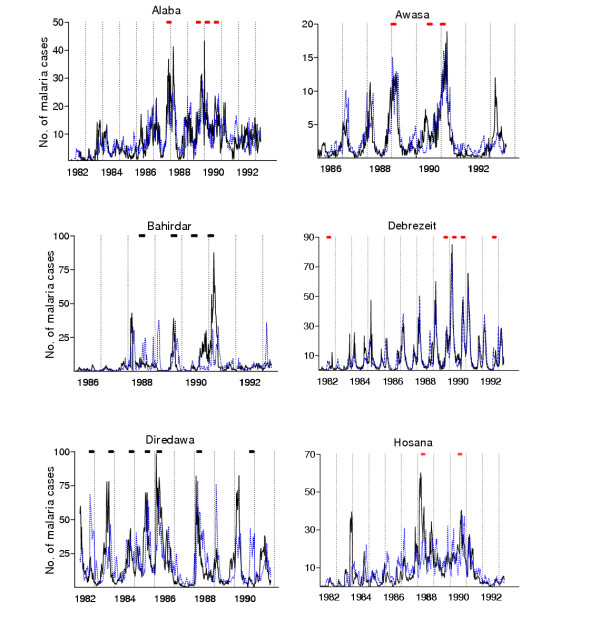
Observed and predicted number of malaria cases with alerts triggered by mean plus 1.5 SD using predicted cases. The solid lines for observed cases and the dotted lines for predicted cases. The red marks are the timing of alerts triggered using predicted cases; their position along the y-axis does not have a meaning.

The prediction algorithm indicates the overall pattern of cases well, yet underestimates the height of the largest peaks. Comparisons of the predicted and observed malaria cases, for each week in six of the ten districts, are shown in Figure 1. The model predicted the actual cases well, although the agreement between the observed and predicted cases varied from district to district. However, the models were not able to differentiate clearly between years with very high and moderately high peaks. To explore whether the predicted number of malaria cases using weather factors can accurately identify time periods with increased number of malaria cases, the timing of alerts triggered, for example, by a mean plus 1.5 standard deviation threshold algorithm, is presented in the same figure. Despite the fact that the actual height of peaks in the highest-incidence periods is poorly predicted by the model, the model nonetheless often triggered alerts prior to these high-incidence periods.

**Figure 2:**
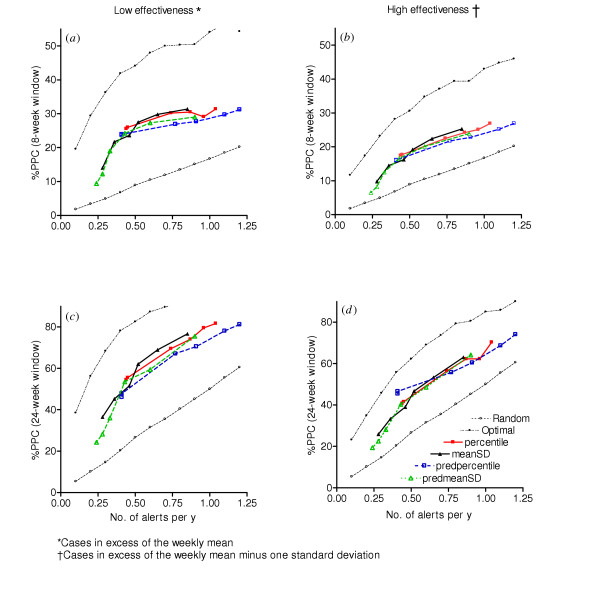
Comparing performance of prediction and detection systems. Percent of PPC by number of alerts per year for different algorithms. (a) and (c) were obtained from cases in excess of the weekly mean (low effectiveness) with window of effectiveness of 8 and 24 weeks respectively. (b) and (d) were obtained from cases in excess of the weekly mean minus one standard deviation (high effectiveness) for windows of eight & 24 weeks, respectively. The solid lines are for detection (Obs) and the dotted lines for prediction (Pred). MeanSD and Percentile represent threshold algorithms based on mean plus standard deviation and percentile, respectively.

The prediction system generates alerts that could prevent nearly as many cases as alerts generated by a detection system. To obtain a quantitative estimate of the usefulness of the prediction algorithm as an early warning system, the %PPC obtained from alerts triggered by predicted cases were compared with %PPC obtained from alerts based on observed cases (Figure 2) under an early detection scheme similar to that previously analyzed [14]. Percentile and mean + standard deviation thresholds are shown, with each point representing a particular value of the threshold (e.g., 85^th ^percentile or mean + 1.50 standard deviations). The horizontal axis gives the number of alerts per year triggered by the particular threshold value, while the vertical axis shows the %PPC associated with that threshold value. Each point represents the mean across all 10 districts. Two different choices of the function for determining PPC (reducing cases to weekly mean: low-effectiveness, a and c, or weekly mean minus one s.d.: high-effectiveness, b and d) and the choice of window of effectiveness (eight weeks, a and b; 24 weeks, c and d) were considered. The performance of the predicted number of malaria cases using the mean plus (0.5, 1, and 1.5) standard deviation algorithm (for an eight-week window of low-effectiveness) reveals that it prevented 29%/0.9 alerts, 27.3%/0.6 alerts and 24.2%/0.43 alerts per year, which compares with 31.4%/0.85 alerts, 29.8%/0.65 alerts and 27.5%/0.52 alerts per year respectively when the observed cases are used to trigger alerts (Figure 2a). In general, relative to alerts triggered by observed cases, the alerts triggered by the predicted number of malaria cases performed slightly worse, within 5% of the detection system. All alerts triggered by predicted and observed cases potentially prevented larger numbers of cases than random alerts. Relative to the optimally timed alerts, both systems performed well, within 10%–20% of the best achievable performance. On average, the number of alerts per year triggered by the prediction system is less than the number of alerts triggered by the observed cases for the corresponding level of alert threshold. Comparative performance of the detection and prediction methods was insensitive to the length of the window of effectiveness and the choice of function to define potentially prevented cases (Figure 2).

**Figure 3:**
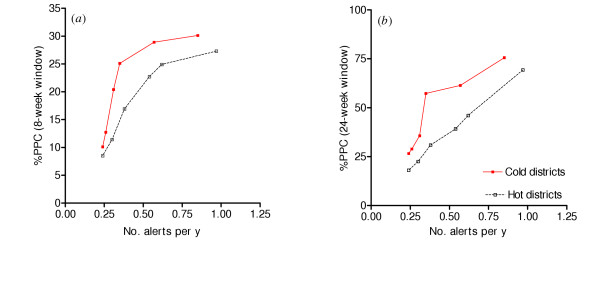
Comparison of performance of prediction systems in cold and hot districts. Percent of PPC by number of alerts per year. PPC was obtained from cases in excess of the weekly mean (low effectiveness) with windows of effectiveness of eight weeks (a) and 24 weeks (b). The solid lines represent cold and the dotted lines hot districts.

Prediction-based systems perform much better in cold than in hot districts. To compare the relative importance of weather factors in cold and hot districts, the %PPC obtained from predicted cases in the cold and hot districts were evaluated separately. Figure 3 shows that alerts triggered by the predicted number of malaria cases in the cold districts perform much better than in the hot districts. Comparative performance in the cold and hot districts was insensitive to the length of the window of effectiveness and the choice of function to define potentially prevented cases. In all cases, the prediction-based alerts were able to prevent 10–25% more cases of malaria at a given sensitivity in cold districts than in hot ones. On the other hand, although, the performance of the detection algorithms in the cold and hot districts was similar with eight-week window of effectiveness, it performed better in the cold than in hot districts with 24-week effectiveness (not shown).

## Discussion

Timely and accurate information about the onset of *P. falciparum *epidemics is essential for effective control activities in epidemic-prone regions, especially those in which limited resources must be deployed to the areas of greatest need. In the Ethiopian highland fringe region, one such epidemic-prone area, early detection of epidemics based on simple algorithms for detecting excess cases had been shown to generate alerts that are well timed to precede periods of high incidence [14]. Early warning methods that provide earlier alerts may allow the interruption of transmission earlier in the epidemic, but perhaps at the expense of some level of accuracy. In this study, we have shown that predictions four weeks ahead, based on weather factors and past case numbers, can provide alerts that are of comparable value to those provided by an equivalent early detection system, based simply on observed cases.

An interesting feature of the results was that the prediction system performed well in generating alerts for control measures, despite the fact that the model under-predicts high peaks. Correlation analyses (data not shown) indicate that for most (but not all) districts, the model performed well qualitatively, in the sense of predicting more cases than expected from the weekly mean when such excess cases occurred, and predicting fewer when in fact fewer cases than the weekly mean occurred. This finding focuses attention on the fact that a system can give timely and accurate alerts for epidemic control, even if it is unable to provide accurate predictions of case numbers (Figure 1). The initial hypothesis was that the improved timeliness of an early detection system comes at the expense of some accuracy. The overall results show that these two effects nearly balance each other, so that early warning systems based on our predictive model provide alerts whose value in terms of epidemic control is comparable to those provided by equivalent early detection systems. In a separate analysis (not shown), these two effects were separated out. If the alert system is based on prediction, but the alerts are timed such that their effects start eight weeks after the alert (i.e., four weeks after the week in which the predicted cases cross the alert threshold, equivalent to the timing for early detection), they identify 5% to 10% fewer PPC than the equivalent detection algorithm. The main analysis (Figure 2) showed that the additional four weeks of notice available by implementing control measures so that their effects begin by the week on which excess cases are predicted (four weeks earlier than if the detection algorithm were used) nearly makes up for this deficit.

Studies have shown that temperature affects transmission in cold environments more than it does in hot environments [31,32]. Thus the addition of minimum and maximum temperature into the prediction model contributes less to predictions in the hot districts than it does in the cold districts. The study revealed this differential effect of weather on malaria transmission. The weather-based prediction system performed much better in the cold than the hot districts. Two mechanisms could have been responsible for this difference: epidemic alert algorithms in general could be less useful in hot districts, or weather-based algorithms, specifically, may be less useful in hot districts. Since simple detection-based alerts performed similarly in hot and cold districts (at least with an eight-week window of effectiveness), it appears that the problem in hot districts is with prediction-based methods. However, when 24 weeks were used as the window of effectiveness, the early detection system, like the prediction system, performed better in the cold than the hot districts. This may be because of the shorter transmission season in the hot than cold districts, due to evaporation and drying up of breeding sites in hot districts, such that rainfall's effects on transmission last for fewer weeks in hot areas [25]. In conclusion, an early warning system using weather and other predictor variables is more reliable in relatively cold than hot districts.

Non-climatic factors such as population immunity, migration and drug resistance are believed to influence malaria transmission and have been cited as causes of malaria epidemics [33-36]. The variability in accuracy of prediction seen in the ten districts may have been due to such factors and others [37-41]. These findings are consistent with the findings of Zhou et al. which indicated that there was high spatial variation in the sensitivity of malaria outpatient number to climate fluctuations in East African highlands [30]. Determining the relative contribution of the non-climatic factors would be an important step in the development of an early warning system for malaria and a predictive model which incorporates such indicators would give more accurate predictions, but this is not feasible in practice at this moment due to the absence of quantitative data on these factors.

The model chosen for the prediction of malaria cases was based loosely on a model previously evaluated for its ability to explain seasonal variation in malaria incidence in the same data set [25]. The former model, in turn, used polynomial distributed lags of weather factors based on biological considerations about the effects of these weather factors on malaria cases. To that model, additional terms – an autoregressive term and an indicator variable for the week of the year (on the Ethiopian calendar) were added – to improve predictive power. The usefulness of this predictive model has been shown, but modifications of the model have not been systematically explored which might improve its predictive ability still further. Further work should consider a range of prediction models for their ability to generate accurate and timely alerts.

## Conclusions

This study showed that short-term (four-week-ahead) predictions of *P. falciparum *cases using lagged weather and case incidence data performed well in identifying periods of increased malaria cases. Furthermore, the prediction system allowed recognition of epidemic periods at an early stage, thereby facilitating interventions making epidemics preventable with adequate lead time. However, this study indicated that early warning system using weather and other predictor variables are more reliable in relatively cold than hot districts. In conclusion, it has been demonstrated that weather derived predictions identified epidemics with reasonable accuracy and better timeliness compared to early detection systems. Therefore, warning systems based on predictions derived from lagged weather variables may be a useful alternative to early detection systems for targeting resources against incipient falciparum malaria epidemics.

## Authors' contributions

HDT, ML and JS conceived the study. HT and ML undertook statistical analysis. HT drafted the manuscript, which was revised by ML. JS participated in designing of the study and statistical analysis. AT initiated the study and made data available in collaboration with WHO and Ministry of Health of Ethiopia. All authors contributed to the writing of the manuscript and approved the submitted version of the manuscript.
